# CALENDAR

**DOI:** 10.1054/bjoc.2000.1693

**Published:** 2001-02


					5-6 March 2001
Second International Conference on Cancer and the
Adolescent
Royal College of Physicians, London, UK
Further information from:
Conference Administrator, Samantha Greshoff. Tel: +44 (0) 20
8333 6311; Fax: +44 (0) 20 8856 3017;
E-mail: sam@greshoff.free-online.co.uk
13-14 March 2001
National Cancer Nursing Conference
Commonwealth Institute,
London, UK
Further information from:
Conference office, Emap Healthcare Events, Greater London
House, Hampstead Road, London NW1 7EJ, UK.
Tel: +44(0) 20 7874 0294; E-mail: conference@healthcare.emap.
co.uk; Website: http://www.nursingtimes.net/shop/shop_index.asp
24-26 May 2001
First International Symposium on Target Volume
Definition in Radiation Oncology
Limburg/Lahn, Germany
Further information from:
Assoc. Prof. Dr IC Kiricuta, Institute for Radiation Oncology,
St. Vincenz-Hospital, Limburg/Lahn, Germany.
Tel: +49 6431 292 4598; Fax: +49 6431 292 4163
1-4 July 2001
British Cancer Research Meeting 2001
University of Leeds, UK
Closing date for abstract submission: 16 February 2001
Further information from:
BCRM Secretariat, c/o Institute of Cancer Research,
Cotswold Road, Sutton, Surrey SM2 5NG, UK.
Tel: +44(0) 20 8722 4208; Fax: +44(0) 20 8770 1395;
E-mail: bcrm@icr.ac.uk
13-16 September 2001
Fifth Leeds International Germ Cell Tumour Conference
GCTC V
Devonshire Hall, University of Leeds, UK
Major topics will include:
* Genetics and Biology of GCTs
* Female GCTs
* Current Status in Testicular GCTs
* Practice Guidelines
Further information from:
Susan Lacey, The University Conference Office, University of
Leeds, Leeds LS2 9JT, UK.
Tel: +44(0) 113 233 6106; Fax: +44(0) 113 233 6107;
E-mail: S.Lacey@Leeds.ac.uk
CALENDAR
British Journal of Cancer (2001) 84(4), 586
(c) 2001 Cancer Research Campaign
doi: 10.1054/ bjoc.2000.1693, available online at http://www.idealibrary.com on
586

				

